# Randomized controlled trial on corneal denervation, neuroinflammation and ocular surface in corneal lenticule extraction for advanced refractive correction (CLEAR) and small incision lenticule extraction (SMILE)

**DOI:** 10.1186/s40662-025-00429-1

**Published:** 2025-04-01

**Authors:** Mingyi Yu, Chang Liu, Isabelle Xin Yu Lee, Victor Wei-Tse Hsu, Regina Kay Ting Wong, Ansa Anam, Rong Lim, Jodhbir S. Mehta, Yu-Chi Liu

**Affiliations:** 1 Tissue Engineering and Cell Therapy Group, Singapore Eye Research Institute, The Academia, 20 College Road, Discovery Tower, Singapore, S169856 Singapore; 2 Department of Ophthalmology, MTI Khyber Teaching Hospital, Peshawar, Pakistan; 3 Lee Kong Chian School of Medicine, Nanyang Technological University, Singapore, Singapore; 4 Department of Cornea and External Eye Disease, Singapore National Eye Centre, Singapore, Singapore; 5 Duke-NUS Medical School, Ophthalmology and Visual Sciences Academic Clinical Program, Singapore, Singapore

**Keywords:** Corneal lenticule extraction for advanced refractive correction (CLEAR), Small incision lenticule extraction (SMILE), Corneal denervation, Neuromediator, Neuroinflammation, Ocular surface

## Abstract

**Background:**

To investigate and compare the corneal denervation, tear neuromediators, and ocular surface changes following corneal lenticule extraction for advanced refractive correction (CLEAR) versus small incision lenticule extraction (SMILE).

**Methods:**

In this randomized clinical trial, 19 patients were randomized to undergo CLEAR in one eye and SMILE in the other eye. Ocular surface assessments, in vivo confocal microscopy for seven corneal nerve parameters, four corneal dendritic cell parameters, three corneal epithelial parameters, and tear neuromediator analysis were performed preoperatively and 1, 3, 6 and 12 months postoperatively.

**Results:**

There were no significant differences in all ocular surface assessments between CLEAR and SMILE throughout postoperative 1 year. CLEAR and SMILE led to significant and comparable reductions of corneal nerve fiber density (CNFD), nerve branch density, total branch density, nerve fiber length, area, and fiber fractal dimension, which did not restore even at 1 year. The reduction in CNFD was significantly correlated with the corrected spherical equivalent in both surgical types. Although post-SMILE eyes had significantly higher nerve growth factor concentrations at 1 month, there was no significant difference in substance P and calcitonin gene-related peptide (CGRP) concentrations between SMILE and CLEAR.

**Conclusions:**

CLEAR and SMILE had comparable effects on ocular surface, corneal denervation and postoperative neuroinflammation. Corneal nerve metrics did not restore even at 1 year for both procedures.

***Trial registration number:*** ClinicalTrials.gov NCT06774651, registration on 14 January 2025, https://clinicaltrials.gov/study/NCT06774651.

**Supplementary Information:**

The online version contains supplementary material available at 10.1186/s40662-025-00429-1.

## Background

Keratorefractive lenticule extraction (KLEx) has garnered significant popularity for correcting myopia and myopic astigmatism over the past two decades [1]. Several types of KLEx procedures have been developed over time [2]. Since its introduction in 2007, small incision lenticule extraction (SMILE), performed by the VisuMax femtosecond laser system (Carl Zeiss Meditec, Jena, Germany), has been shown to yield equivalent clinical safety, efficacy, predictability, and stability compared with laser-assisted in situ keratomileusis (LASIK) [3]. Furthermore, several studies have confirmed the superiority of SMILE in terms of corneal nerve preservation, impacts on the ocular surface, and postoperative neuroinflammation [4–6].

In 2020, the corneal lenticule extraction for advanced refractive correction (CLEAR) procedure, delivered on the Femto LDV Z8 platform (Ziemer Ophthalmic Systems AG, Port, Switzerland), received the Conformité Européenne approval [7]. The Femto LDV Z8 is a low-energy and high-frequency system [8], achieving photo-disruption in the low nanojoules range (< 100 nJ) [7, 9]. With minimal stromal gas generation, it thereby reduces the risk of opaque bubble formation and unsuccessful lenticule dissections [7]. Another advantage of the CLEAR procedure is that it allows the creation of two corneal incisions and two guiding tunnels, allowing the surgeons to dissect the anterior and posterior surface of the lenticule respectively, shortening the learning curve. This may also decrease extensive intrastromal manipulations and mechanical disruption of the corneal tissue during the procedure. Leccisotti et al. reported the visual and refractive outcomes of 78 eyes following the CLEAR procedure [10]. The results demonstrated that 75% of eyes achieved an uncorrected distance visual acuity (UCVA) of 20/25 or better, while 85% of eyes were within ± 0.50 diopters (D) of the target spherical equivalent (SE). However, there is a lack of studies investigating the degree of corneal denervation and regeneration, neuroinflammation, and alterations in the ocular surface following CLEAR.

It has been known that corneal denervation and resultant neuroinflammation due to refractive surgery can lead to negative impacts on the ocular surface, causing dry eye [11]. Long-lasting dry eye symptoms after refractive surgery are an important cause of patient dissatisfaction [12]. Disruption of corneal nerve plexuses impairs the nerve-derived neurotrophic factors and corneal sensation, resulting in decreased tear secretion, abnormal cornea-blink reflex, and alterations in tear film dynamics [13, 14]. The intraoperative application of a suction ring may also temporarily increase the pressure on the conjunctiva, causing goblet cell damage and contributing to postoperative tear dysfunction [15]. Several studies have demonstrated the changes in proinflammatory neurotransmitters and neuropeptides in tears following refractive surgery, with substance P, calcitonin gene-related peptide (CGRP), and nerve growth factor (NGF) being the most investigated neuromediators [5, 11]. We have also previously demonstrated that following SMILE, the 1-month tear substance P, and CGRP concentrations were significantly correlated with the corrected SE [16]. However, reports on postoperative neuroinflammation following other types of KLEx procedures, such as CLEAR, are still lacking. Furthermore, Femto LDV Z8 and VisuMax systems differ in laser pulse energy, contact interface curvature, suction area and suction time, and lenticule profile. The impact of these factors on postoperative corneal denervation, neuroinflammation, and ocular surface outcomes remains unclear.

In this randomized controlled trial (RCT), we aimed to investigate and compare the ocular surface changes, corneal denervation, and nerve regeneration, as well as tear neuromediator profiles in CLEAR versus SMILE in a 1-year study. This study will provide a better understanding of the corneal alterations in CLEAR versus SMILE on a cellular and molecular level, as well as the long-term implications and differences between these two procedures.

## Methods

### Study design and population

This was a single-masked, paired-eye design, prospective randomized clinical trial that included 19 patients, randomized to undergo CLEAR in one eye and SMILE in the other eye between September 2021 and April 2023 at the Singapore National Eye Center. The inclusion and exclusion criteria are listed in Supplementary Table 1. Patients’ characteristics including age, sex, and preoperative manifest refractive spherical equivalent (MRSE) were collected. The random allocation sequence was performed by a random number generator, with no blocks or restrictions, and implemented by concealing the number-coded surgery within sealed envelopes until just before the procedure. Each subject underwent either CLEAR or SMILE in one eye, followed by SMILE or CLEAR in the fellow eye on the same day (either the left or right eye was randomized to decide which eye was operated on first). Both the participants and the outcome assessors were masked to the procedure. Approval for the study was granted by the Institutional Review Board of SingHealth, Singapore (No. 2021/2575). The study was conducted in accordance with the Declaration of Helsinki and informed consent was obtained from all participants.

### Surgical procedures

CLEAR procedure was performed using the Femto LDV Z8 laser. The patients were instructed to fixate on the fixation light, and suction was initiated. A flat contact glass of suction system was used. The laser power percentage scale used were 41%–51% and 44%–55% for the creation of the anterior and posterior lenticule surfaces, respectively. The cap thickness was set between 110 and 130 µm, the optical zone was 6.0 to 6.5 mm, and the guiding tunnel was 0.4 to 0.6 mm. The single 2.1 mm incision was created at 140° position with an entrance angle of 90 degrees. Following laser photodisruption, the anterior and posterior lenticular interfaces were separated using a lamellar dissector (ASICO, Westmont IL, USA). The lenticule was then removed through the small incision with Tan EndoGlide forceps (AngioTech, Network Medical Products). The SMILE procedure was performed as previously described [17, 18]. An S-sized curved cone was applied, and the Visumax femtosecond laser (VisuMax500) with the power set at 145 nJ was used. The cap thickness was 100 to 130 μm, the cap diameter was 7.5 mm, the optical zone was 6.0 to 6.5 mm, and the side-cut angle of 90° was used. A 2.1 mm vertical circumferential incision was placed at 120°. A lamellar dissector was used for the dissection of the lenticule, and then the lenticule was grasped and removed using Tan EndoGlide forceps. The instruments for lenticule dissection and extraction are identical between CLEAR and SMILE. The optical zone, cap thickness, and incision size for the CLEAR and the SMILE eye were identical for the same patient. All procedures used topical anesthesia and were performed by the same refractive surgeon (JSM). The postoperative regimen for CLEAR and SMILE groups was identical, consisting of topical preservative-free dexamethasone (Maxidex, Alcon Laboratories, Inc.) and moxifloxacin (Vigamox, Alcon Laboratories, Inc.), 3 hourly for 1 week and then 4 times daily for 2 weeks. Subsequently, artificial tears (Tears Naturale Free; Alcon Laboratories, Inc.) were prescribed with a frequency of 4 times daily for the first 3 months and then adjusted based on the patients’ symptoms.

### Ocular surface examinations

Assessment of the ocular surface was performed in all patients preoperatively and at 1 week, and 1, 3, 6 and 12 months postoperatively, as previously described protocols [5, 19]. The assessments include Schirmer’s test I (assessed without topical anesthesia for 5 min), corneal fluorescein staining [National Eye Institute (NEI) scale; 0: minimal, 15: maximal], ocular surface fluorescein staining (Oxford grading; 0: absent, 5: severe), tear break-up time (TBUT), and corneal sensitivity (Cochet-Bonnet aesthesiometer, Ophtalmologie, Chartres, France; 0 to 6 cm for each of the 4 quadrants and the central cornea, 0 to 30 cm for the whole cornea). All examinations were performed by an ophthalmologist (CL). Three consecutive measurements were taken at each visit, and the average of the measurements was used for data analysis.

### In vivo confocal microscopy (IVCM) image acquisition and analysis

The Heidelberg HRT3 Rostock Cornea Module (Heidelberg Engineering GmbH, Heidelberg, Germany) was used for the subbasal corneal nerve plexus scans preoperatively and at 1, 3, 6, and 12 months postoperatively using IVCM by two experienced masked operators with the protocol as previously described [4, 20]. After topical anesthesia, the patients were instructed to fixate on a flashing light in different directions with the contralateral eye to stabilize the scanning view. Images were taken of the central cornea, and the nasal, temporal, superior, and inferior quadrants approximately 3 mm from the corneal apex, covering a 400 × 400 µm field of view [21].

For each IVCM scanned area, five best-focused and most representative images of subbasal nerves were selected. All images were evaluated using the automatic analysis software ACCMetrics (University of Manchester, Manchester, UK) with the following seven parameters quantified: corneal nerve fiber density (CNFD; number of main nerve fibers/mm^2^), corneal nerve fiber length (CNFL; the total length of fibers in mm/mm^2^), corneal nerve branch density (CNBD; number of branch points on the main fibers/mm^2^), corneal nerve total branch density (CTBD; total number of branch points/mm^2^), corneal nerve fiber area (CNFA; total nerve fiber area mm^2^/mm^2^), corneal nerve fiber width (CNFW; mean nerve fiber width in mm/mm^2^), and nerve fiber fractal dimension (CFracDim, measurement of the spatial loss of corneal nerves, where a high CFracDim value indicates an evenly distributed complex nerve fiber structure) [22, 23]. Nerve parameters at 1 month postoperatively were used to study the correlation between denervation and ocular surface changes as early postoperative nerve parameters are less affected by nerve regeneration activity and more representative of the state of corneal denervation. The extent of denervation was represented by the percentage of corneal reduction, calculated as $1-\frac{\left(1-{\text{month parameters}}\right)}{{\text{preoperative parameters}}}\left({\%}\right)$[24].

For the analysis of the corneal epithelial cells, three most representative images with clear epithelial cell borders and a large area of coverage were selected. The AlConfocal Rapid Image Evaluation System (ARIES; ADCIS, France) automated software was used for the quantification of corneal epithelial cells, as described previously [20]. Epithelial cell morphology measurements included cell density (cells/mm^2^), average size (μm^2^), and circularity. Cell circularity represents how closely the shape of cells approaches that of a circle, and a circularity value of 1.0 indicates an ideal circle [25].

Corneal dendritic cells (DCs) were also analyzed with the ARIES software based on the three most representative micro-images. DCs were quantified with these four parameters: cell density (cells/mm^2^), area of cells (μm^2^), elongation [calculated as the absolute value of the difference between the major and minor axes divided by the sum of the major and minor axes (µm)], and the average cell length (μm) [26].

Corneal microneuromas, characterized by irregularly shaped enlargements of terminal nerve endings with poorly defined margins and variable hyper-reflectivity, were manually identified [27]. The images containing corneal microneuromas were manually quantified using Image J software (NIH, USA). The parameters assessed included the microneuroma area (µm^2^) and perimeter (µm). All the images were analyzed by a single experienced masked investigator.

### Tear neuromediator analysis

Tear samples were collected using Schirmer strips, cut into small pieces, and submerged in 200 µL of an ice-cold tear elution buffer containing 0.55 M NaCl, 0.33% Tween-20, 0.55% bovine serum albumin, and 1X protease inhibitor, followed by sonication and homogenization and incubation at 4 °C with gentle agitation for 17 h [16]. The supernatants were collected after centrifugation for protein analysis. Enzyme-linked immunosorbent assay was subsequently performed according to the manufacturer’s protocol (CGRP from Phoenix Pharmaceuticals, Runcorn, UK; Substance P and NGF from R&D Systems, Minneapolis, USA). The eluted tear samples were diluted to 50 µL per well with dilution factors of 4, 4, and 1.5 for substances P, CGRP, and NGF, respectively [28].

### Sample size calculation

The required sample size was calculated based on the pilot data of tear NGF concentrations at 1 month from five patients: 30.18 ± 10.86 pg/mL in CLEAR and 20.98 ± 11.45 pg/mL in SMILE. Hence, a sample size of 17 patients, with a power of 80% and at a 5% level of significance, was sufficient to detect differences between the two groups.

### Statistical analysis

All data were expressed as mean ± standard deviation. The comparisons between the CLEAR and SMILE eyes were performed using an independent *t* test. The comparisons between the postoperative follow-up and baseline in both CLEAR and SMILE group eyes were performed using repeated measures ANOVA followed by Bonferroni post-hoc tests. Statistical analyses were performed with SPSS version 26 (IBM SPSS Statistics, Chicago, IL, USA). Differences between the groups with *P* values of less than 0.05 were considered significant.

## Results

### Patient characteristics

The mean age of patients was 32.97 ± 7.59 years (range, 23–49 years). Among them, 10 (52.63%) were female. CLEAR surgery was performed on the right eye of 11 patients (57.89%) and on the left eye of 8 patients (42.11%). There were no significant differences between CLEAR and SMILE eyes in the preoperative MRSE, programmed SE correction, logMAR UCVA, and logMAR best-correct visual acuity (BCVA). Table 1 summarizes the detailed preoperative and intraoperative characteristics.

**Table 1:** Preoperative and intraoperative characteristics of the study populations

Parameter	CLEAR	SMILE	*P* value
Number of eyes (patients)	19 (19)	19 (19)	–
Gender, n (%)			
Male	9 (47.37)		–
Female	10 (52.63)		–
Age (years), (range)	32.97 ± 7.59 (23–49)		–
Eye laterality, n (%)			
Right	11 (57.89)	8 (42.11)	–
Left	8 (42.11)	11 (57.89)	–
UDVA (logMAR)	1.10 ± 0.15	1.16 ± 0.15	0.243
BCVA (logMAR)	− 0.04 ± 0.03	− 0.04 ± 0.04	0.699
Preoperative MRSE (D)	− 5.90 ± 2.14	− 6.11 ± 2.01	0.703
Programmed SE correction (D)	− 6.48 ± 2.32	− 6.79 ± 2.25	0.821
Incision number	1	1	–
Incision size (mm)	2.1	2.1	–
Optical zone diameter (mm)	6.4 ± 0.2	6.4 ± 0.2	–
Spherical equivalent correction (D)	− 6.5 ± 2.3	− 6.3 ± 2.5	0.821
Cap thickness (μm)	114.6 ± 6.9	112.7 ± 9.1	0.601
Lenticule thickness (μm)	120.5 ± 29.9	119.2 ± 32.3	0.920
Residual stroma thickness (μm)	322.9 ± 34.7	332. 5 ± 30.1	0.499

*CLEAR* = corneal lenticule extraction for advanced refractive correction; *SMILE* = small incision lenticule extraction; *MRSE* = manifest refractive spherical equivalent; *UDVA* = uncorrected distance visual acuity; *BCVA* = best-corrected visual acuity

### Ocular surface assessments

There were no significant differences between the CLEAR and SMILE groups with regards to corneal sensitivity, Schirmer’s test, TBUT, Oxford and NEI scores preoperatively or at any postoperative time points. Compared to preoperative levels, TBUT was significantly decreased at the 1-week timepoint in CLEAR eyes (*P* = 0.003) and significantly decreased by 1 month in SMILE eyes (*P* < 0.001; Table 2).

**Table 2:** Ocular surface assessment in CLEAR vs. SMILE

Parameter	CLEAR	SMILE	*P* value^a^	*P* value^b^	*P* value^c^
Preoperative					
Corneal sensitivity (mm)	29.61 ± 1.25	28.19 ± 5.93	0.317	–	–
Schirmer’s test value (mm)	16.94 ± 12.26	15.83 ± 11.31	0.779	–	–
TBUT (s)	7.39 ± 2.66	7.42 ± 2.46	0.975	–	–
Oxford Score	0.16 ± 0.37	0.11 ± 0.32	0.642	–	–
NEI Score	0.32 ± 0.67	0.32 ± 0.58	1.000	–	–
Postoperative week 1					
Corneal sensitivity (mm)	26.25 ± 8.50	26.25 ± 7.78	1.000	0.124	0.168
Schirmer’s test value (mm)	14.80 ± 11.91	14.13 ± 13.00	0.885	0.718	1.000
TBUT (s)	5.61 ± 2.56	5.95 ± 2.50	0.679	**0.003**	**0.009**
Oxford Score	0.26 ± 0.56	0.21 ± 0.42	0.745	0.429	0.429
NEI Score	0.84 ± 1.50	0.37 ± 0.76	0.228	0.066	0.749
Postoperative month 1					
Corneal sensitivity (mm)	28.94 ± 2.016	28.69 ± 2.36	0.749	0.383	0.611
Schirmer’s test value (mm)	12.33 ± 8.90	14.22 ± 11.32	0.582	0.067	0.501
TBUT (s)	5.78 ± 2.21	5.26 ± 2.42	0.505	**0.034**	** < 0.001**
Oxford Score	0.16 ± 0.50	0.05 ± 0.23	0.411	1.000	0.578
NEI Score	0.76 ± 1.70	0.29 ± 0.51	0.253	0.182	0.881
Postoperative month 3					
Corneal sensitivity (mm)	27.53 ± 4.09	27.53 ± 4.09	1.000	0.054	0.722
Schirmer’s test value (mm)	15.42 ± 12.69	16.47 ± 11.52	0.801	0.284	0.918
TBUT (s)	7.68 ± 2.37	6.69 ± 2.12	0.204	0.698	0.417
Oxford Score	0.17 ± 0.51	0.11 ± 0.32	0.701	1.000	1.000
NEI Score	0.44 ± 1.65	0.17 ± 0.51	0.501	0.734	0.269
Postoperative month 6					
Corneal sensitivity (mm)	29.06 ± 2.11	28.18 ± 3.11	0.340	0.392	0.959
Schirmer’s test value (mm)	11.50 ± 8.64	11.38 ± 10.16	0.795	0.145	0.164
TBUT (s)	7.11 ± 2.03	7.22 ± 1.99	0.869	0.685	0.712
Oxford Score	0.11 ± 0.32	0.17 ± 0.51	0.701	0.668	0.717
NEI Score	0.22 ± 0.55	0.17 ± 0.38	0.727	0.579	0.269
Postoperative month 12					
Corneal sensitivity (mm)	28.08 ± 4.35	28.08 ± 4.35	1.000	0.316	0.814
Schirmer’s test value (mm)	9.42 ± 8.89	10.83 ± 11.78	0.743	0.093	0.294
TBUT (s)	6.64 ± 1.60	7.00 ± 2.08	0.614	0.676	0.944
Oxford Score	0.29 ± 0.61	0.14 ± 0.53	0.516	0.671	1.000
NEI Score	0.21 ± 0.58	0.14 ± 0.53	0.737	0.385	0.272

*P* values in bold indicate statistical significance

*CLEAR* = corneal lenticule extraction for advanced refractive correction; *SMILE* = small incision lenticule extraction; *TBUT* = tear break-up time; *NEI* = National Eye Institute

^a^CLEAR vs. SMILE eyes

^b^Postoperative vs. preoperative for CLEAR eyes

^c^Postoperative vs. preoperative for SMILE eyes

### Corneal denervation after CLEAR and SMILE

**Fig. 1:**
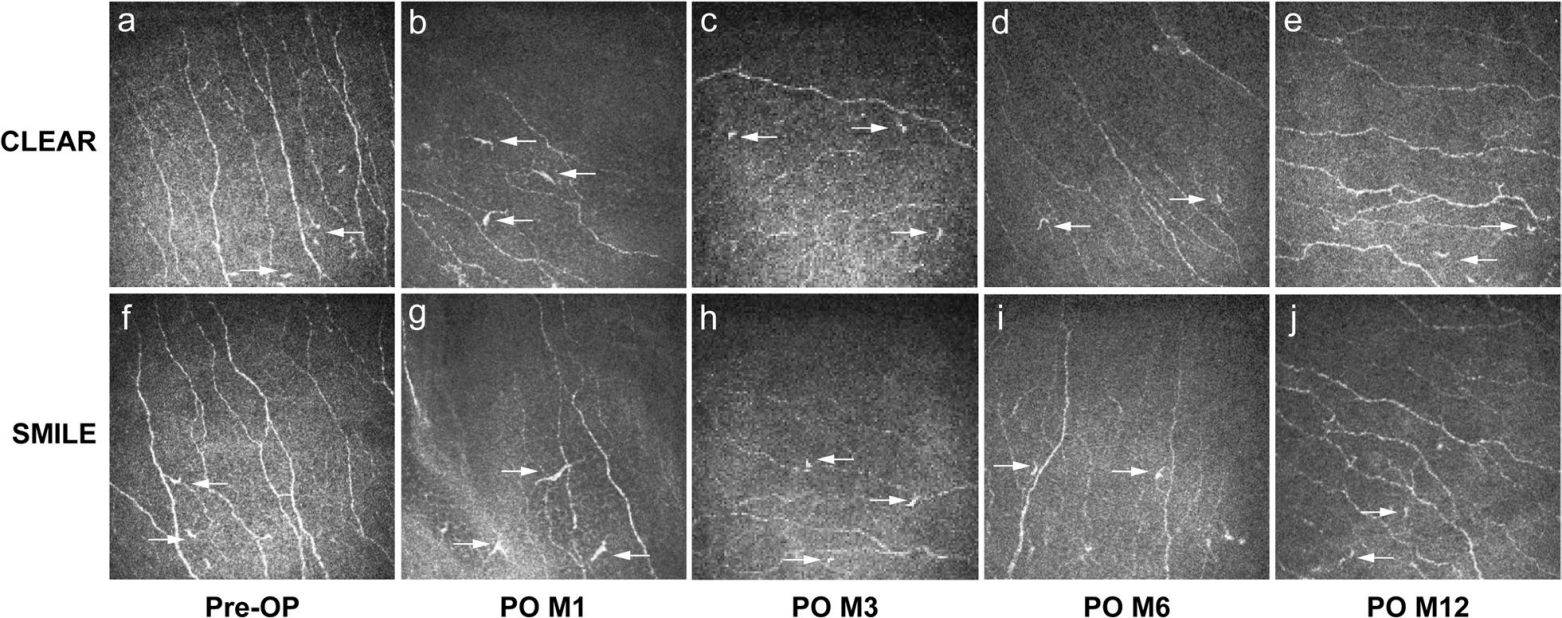
Representative IVCM images of the subbasal nerve plexus from preoperative to 12 months postoperatively. Both CLEAR eyes (**a**–**e**) and SMILE eyes (**f**–**j**) showed significant reductions in CNFD, CNBD, CNFL, CTBD, and CFracDim compared with preoperative levels. Corneal dendritic cells (white arrows) were detected at all time points, presenting with a notable increased cell area at 1 month. IVCM, in vivo confocal microscopy; CLEAR, corneal lenticule extraction for advanced refractive correction; SMILE, small incision lenticule extraction; CNFD, corneal nerve fiber density; CNBD, corneal nerve branch density; CNFL, corneal nerve fiber length; CTBD, corneal total branch density; CFracDim, corneal nerve fiber fractal dimension; Pre-OP, preoperative; PO, postoperative; M, month

Corneal nerve parameters were comparable between the CLEAR and SMILE groups at all time points (Table 3; Fig. 1). Throughout the postoperative 12-month period, statistically significant reductions in CNFD, CNBD, CDFL, CTBD, and CFracDim were observed in both CLEAR and SMILE groups when compared to their respective preoperative levels (all *P* < 0.05), suggesting that the postoperative corneal nerve metrics did not restore to the preoperative level even at 12 months. Reductions in CNFA were significant at postoperative 1, 3, and 6 months (*P* = 0.001, *P* = 0.003, *P* = 0.027, respectively) in both groups.

**Table 3:** Comparison of corneal nerve parameters in CLEAR vs. SMILE

Parameter	CLEAR	SMILE	*P* value^a^	*P* value^b^	*P* value^c^
Preoperative					
CNFD (/mm^2^)	16.11 ± 3.46	18.26 ± 4.06	0.169	–	–
CNBD (/mm^2^)	14.05 ± 4.81	15.46 ± 3.86	0.387	–	–
CNFL (mm/mm^2^)	9.14 ± 2.44	10.59 ± 2.60	0.121	–	–
CTBD (/mm^2^)	20.34 ± 7.68	24.14 ± 9.17	0.228	–	–
CNFA (mm^2^/mm^2^)	0.0044 ± 0.0010	0.0047 ± 0.0011	0.409	–	–
CNFW (mm/mm^2^)	0.0225 ± 0.0019	0.0215 ± 0.0012	0.069	–	–
CFracDim	1.41 ± 0.04	1.43 ± 0.03	0.102	–	–
Postoperative month 1					
CNFD (/mm^2^)	8.30 ± 4.15	8.90 ± 3.53	0.649	**0.013**	** < 0.001**
CNBD (/mm^2^)	5.28 ± 4.89	4.62 ± 3.14	0.643	**0.009**	** < 0.001**
CNFL (mm/mm^2^)	6.59 ± 2.18	6.50 ± 2.16	0.897	**0.039**	** < 0.001**
CTBD (/mm^2^)	11.16 ± 6.06	11.49 ± 5.14	0.865	**0.009**	**0.003**
CNFA (mm^2^/mm^2^)	0.0012 ± 0.0028	0.0009 ± 0.0025	0.795	**0.009**	**0.001**
CNFW (mm/mm^2^)	0.0208 ± 0.0025	0.0207 ± 0.0024	0.876	0.206	0.365
CFracDim	1.36 ± 0.04	1.35 ± 0.05	0.410	**0.027**	** < 0.001**
Postoperative month 3					
CNFD (/mm^2^)	7.08 ± 3.67	8.49 ± 3.45	0.249	** < 0.001**	** < 0.001**
CNBD (/mm^2^)	3.83 ± 2.89	5.26 ± 3.84	0.226	** < 0.001**	** < 0.001**
CNFL (mm/mm^2^)	6.01 ± 1.85	6.51 ± 2.04	0.457	** < 0.001**	**0.001**
CTBD (/mm^2^)	10.54 ± 4.24	11.66 ± 7.26	0.585	**0.001**	**0.003**
CNFA (mm^2^/mm^2^)	0.0029 ± 0.0010	0.0030 ± 0.0012	0.743	**0.001**	**0.003**
CNFW (mm/mm^2^)	0.0220 ± 0.0012	0.0216 ± 0.0011	0.292	0.479	0.969
CFracDim	1.3437 ± 0.0445	1.3536 ± 0.0540	0.556	** < 0.001**	** < 0.001**
Postoperative month 6					
CNFD (/mm^2^)	7.08 ± 3.67	7.68 ± 3.82	0.605	** < 0.001**	** < 0.001**
CNBD (/mm^2^)	3.83 ± 2.89	6.03 ± 4.38	0.593	** < 0.001**	** < 0.001**
CNFL (mm/mm^2^)	6.01 ± 1.85	6.44 ± 2.15	0.829	**0.002**	**0.001**
CTBD (/mm^2^)	11.10 ± 4.74	11.53 ± 4.67	0.804	** < 0.001**	**0.001**
CNFA (mm^2^/mm^2^)	0.0029 ± 0.0010	0.0033 ± 0.0010	0.406	** < 0.001**	**0.027**
CNFW (mm/mm^2^)	0.0220 ± 0.0012	0.0219 ± 0.0011	0.831	0.151	0.286
CFracDim	1.34 ± 0.04	1.35 ± 0.04	0.952	**0.005**	** < 0.001**
Postoperative month 12					
CNFD (/mm^2^)	10.11 ± 2.88	10.71 ± 3.06	0.646	**0.010**	** < 0.001**
CNBD (/mm^2^)	7.11 ± 4.95	6.70 ± 5.35	0.858	**0.036**	**0.001**
CNFL (mm/mm^2^)	7.05 ± 1.57	6.90 ± 1.78	0.837	**0.011**	**0.008**
CTBD (/mm^2^)	12.87 ± 4.17	12.86 ± 7.54	0.997	**0.028**	**0.013**
CNFA (mm^2^/mm^2^)	0.0034 ± 0.0008	0.0031 ± 0.0011	0.433	0.053	0.052
CNFW (mm/mm^2^)	0.0215 ± 0.0007	0.0210 ± 0.0007	0.097	0.852	0.637
CFracDim	1.37 ± 0.03	1.36 ± 0.04	0.547	**0.001**	**0.002**

*P* values in bold indicate statistical significance

*CLEAR* = corneal lenticule extraction for advanced refractive correction; *SMILE* = small incision lenticule extraction; *CNFD* = corneal nerve fiber density; *CNBD* = corneal nerve branch density; *CNFL* = corneal nerve fiber length; *CTBD* = corneal total branch density; *CNFA* = corneal nerve fiber area; *CNFW* = corneal nerve fiber width; *CFracDim* = corneal nerve fiber fractal dimension

^a^CLEAR vs. SMILE eyes

^b^Postoperative vs*.* preoperative for CLEAR eyes

^c^Postoperative vs. preoperative for SMILE eyes

### Correlation between corneal denervation and corrected refractive power

**Fig. 2:**
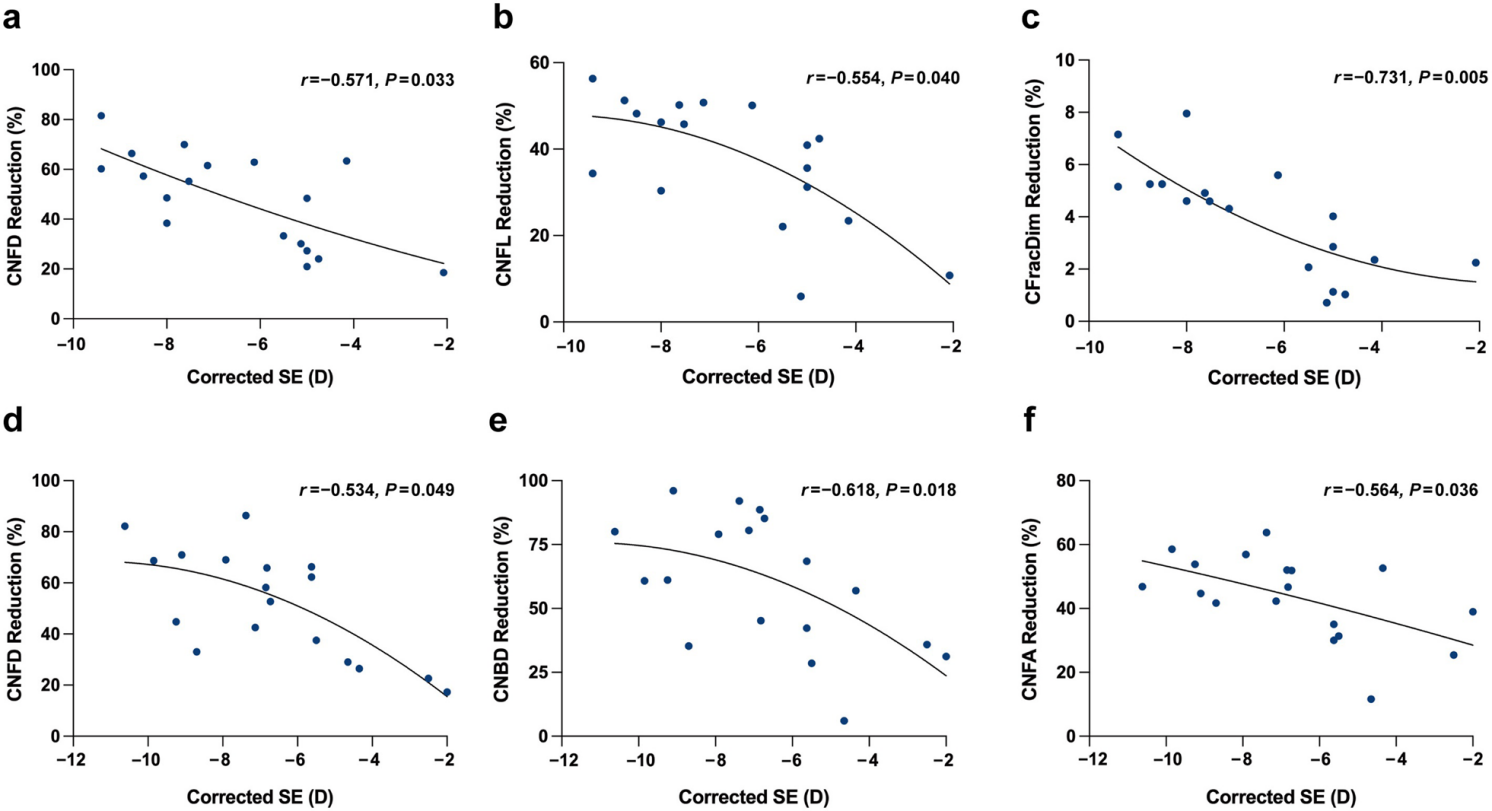
Scatter plots showing the correlation between corrected SE and reductions in corneal nerve parameters. Correlation between the corrected SE and the reduction in CNFD (**a**), CNFL (**b**), and CFracDim (**c**) in the CLEAR eyes. Correlation between the corrected SE and the reduction in CNFD (**d**), CNBD (**e**), and CNFA (**f**) in the SMILE eyes. SE, spherical equivalent; CNFD, corneal nerve fiber density; CNFL, corneal nerve fiber length; CFracDim, corneal nerve fiber fractal dimension; CLEAR, corneal lenticule extraction for advanced refractive correction; CNBD, corneal nerve branch density; CNFA, corneal nerve fiber area; SMILE, small incision lenticule extraction

In the CLEAR group, the corrected SE had a significant and strong correlation with the reduction in CFracDim (*r* =  − 0.731, *P* = 0.005), and a significant and moderate correlation with the reduction in CNFD (*r* =  − 0.571, *P* = 0.033) and CNFL (*r* =  − 0.554, *P* = 0.040; Fig. 2 a-c). In the SMILE group, a significant and moderate correlation was observed between the corrected SE and the reduction in CNFD (*r* =  − 0.534, *P* = 0.049), CNBD (*r* =  − 0.618, *P* = 0.018), and CNFA (*r* =  − 0.564, *P* = 0.036; Fig. 2d–f). These findings indicate that higher refractive treatment led to a greater extent of corneal nerve impairment in both procedures.

### Correlation between corneal nerve parameters and ocular surface assessments

**Fig. 3:**
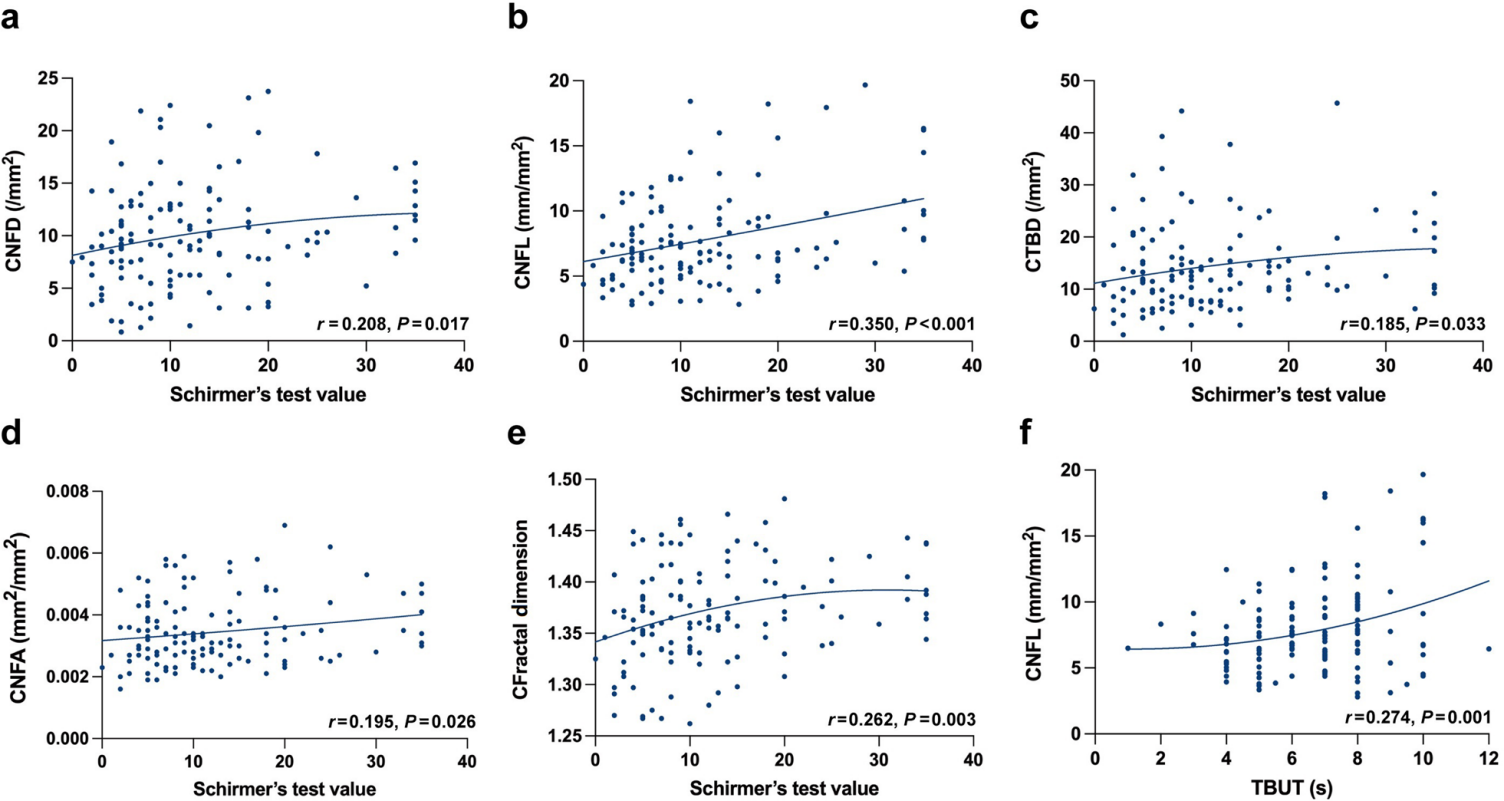
Scatter plots showing the correlation between corneal nerve parameters and ocular surface assessments. Correlation between the Schirmer’s test value and CNFD (**a**), CNFL (**b**), CTBD (**c**), CNFA (**d**), and CFracDim (**e**). Correlation between the TBUT and CNFL (**f**). CNFD, corneal nerve fiber density; CNFL, corneal nerve fiber length; CTBD, corneal total branch density; CNFA, corneal nerve fiber area; CFracDim, corneal nerve fiber fractal dimension; TBUT, tear break-up time

From Fig. 3, there was a significant and positive correlation between Schirmer’s test values and CNFD (*r* = 0.208, *P* = 0.017), CNFL (*r* = 0.350, *P* < 0.001), CTBD (*r* = 0.185, *P* = 0.033), CNFA (*r* = 0.195, *P* = 0.026), and CFracDim (*r* = 0.262, *P* = 0.003). Additionally, TBUT was positively and significantly correlated with CNFL (*r* = 0.274, *P* = 0.001).

### Corneal dendritic cell parameters

In the SMILE group, the area of corneal DCs increased significantly at 1 month (*P* = 0.034), followed by a gradual decline thereafter (Table 4; Fig. 1). The CLEAR group showed no significant changes in all the parameters of DCs at all postoperative time points compared to preoperative levels. There were no significant differences in any of the parameters between the two groups throughout the study period (Table 4).

**Table 4:** Comparison of corneal dendritic cell parameters in CLEAR vs. SMILE

Parameter	CLEAR	SMILE	*P* value^a^	*P* value^b^	*P* value^c^
Preoperative					
Cell density (/μm^2^)	0.0197 ± 0.0018	0.0237 ± 0.0064	0.195	–	–
Area of cell (μm^2^)	52.10 ± 4.99	47.26 ± 11.78	0.376	–	–
Elongation	0.72 ± 0.05	0.67 ± 0.06	0.083	–	–
Average cell length (μm)	13.04 ± 1.59	11.93 ± 2.32	0.123	–	–
Postoperative month 1					
Cell density (/μm^2^)	0.0125 ± 0.0126	0.0125 ± 0.0050	1.000	0.529	0.057
Area of cell (μm^2^)	85.64 ± 62.05	76.75 ± 18.16	0.466	0.216	**0.034**
Elongation	0.70 ± 0.08	0.75 ± 0.07	0.381	0.593	0.285
Average cell length (μm)	23.02 ± 10.96	19.76 ± 4.24	0.599	0.285	0.109
Postoperative month 3					
Cell density (/μm^2^)	0.0183 ± 0.0033	0.0185 ± 0.0030	0.923	0.996	0.494
Area of cell (μm^2^)	57.71 ± 9.98	60.14 ± 15.26	0.773	0.891	0.858
Elongation	0.70 ± 0.07	0.71 ± 0.08	0.929	0.465	0.465
Average cell length (μm)	14.88 ± 2.76	16.04 ± 5.24	0.671	0.273	0.715
Postoperative month 6					
Cell density (/μm^2^)	0.0206 ± 0.0019	0.0222 ± 0.0027	0.277	0.513	0.682
Area of cell (μm^2^)	51.09 ± 6.51	47.70 ± 7.14	0.411	0.440	0.586
Elongation	0.64 ± 0.02	0.65 ± 0.06	0.825	0.109	0.180
Average cell length (μm)	12.61 ± 1.74	11.98 ± 1.69	0.543	0.480	0.285
Postoperative month 12					
Cell density (/μm^2^)	0.0217 ± 0.0013	0.0242 ± 0.0019	0.066	0.060	0.871
Area of cell (μm^2^)	46.64 ± 3.54	42.00 ± 3.22	0.062	0.101	0.362
Elongation	0.63 ± 0.05	0.63 ± 0.03	0.874	0.090	0.269
Average cell length (μm)	11.38 ± 1.16	10.39 ± 0.46	0.113	0.064	0.181

*P* values in bold indicate statistical significance

*CLEAR* = corneal lenticule extraction for advanced refractive correction; *SMILE* = small incision lenticule extraction

^a^CLEAR vs. SMILE eyes

^b^Postoperative vs. preoperative for CLEAR eyes

^c^Postoperative vs. preoperative for SMILE eyes

### Analysis of corneal microneuromas

**Fig. 4:**
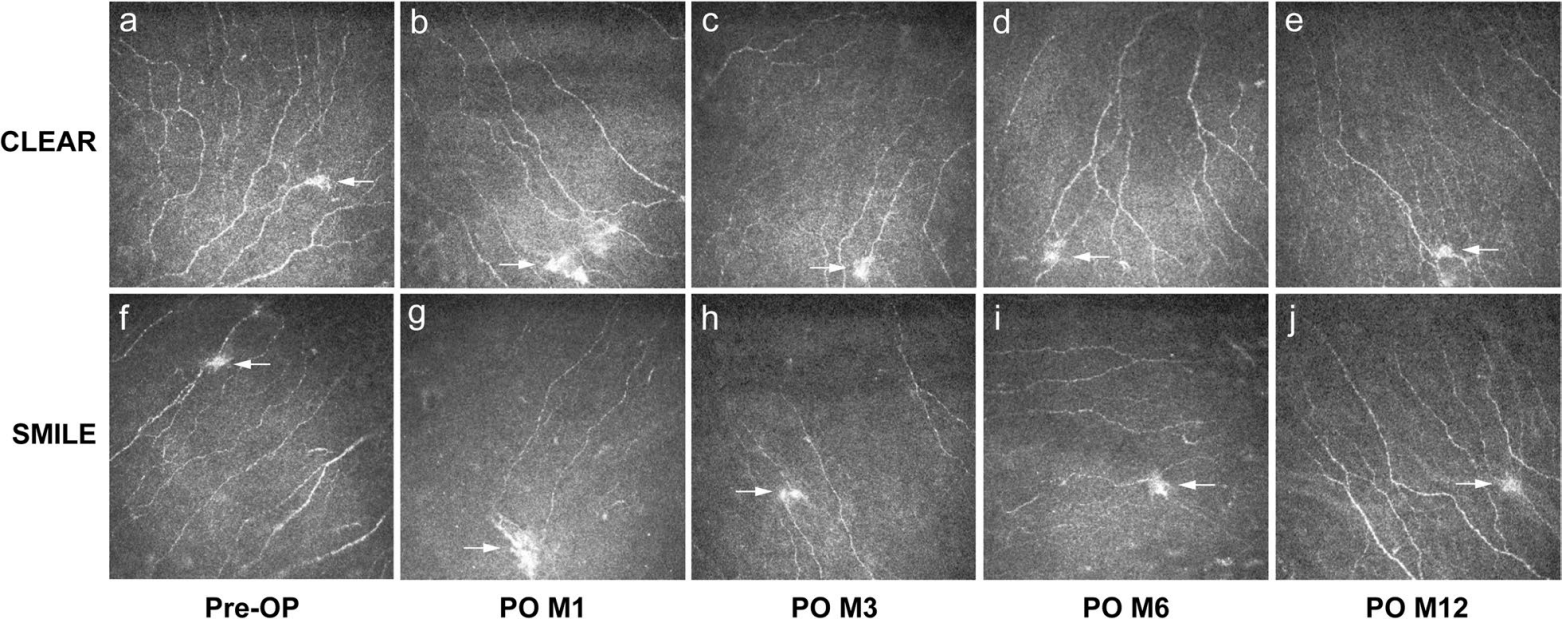
Representative IVCM images of microneuromas. The total area and perimeter of microneuromas were significantly larger at 1 month postoperatively compared to preoperative levels in both CLEAR (**a**, **b**) and SMILE eyes (**f**, **g**), and these measurements gradually decreased over the subsequent study period. IVCM, in vivo confocal microscopy; CLEAR, corneal lenticule extraction for advanced refractive correction; SMILE, small incision lenticule extraction; Pre-OP, preoperative; PO, postoperative; M, month

CLEAR and SMILE eyes exhibited similar patterns regarding the total area and perimeter of the microneuromas with no significant differences throughout the study period (Table 5). Both groups presented a significant increase in the total area and perimeter of microneuromas at 1 month, followed by a gradual decrease over time (Table 5; Fig. 4).

**Table 5:** Comparison of microneuroma parameters in CLEAR vs. SMILE

Parameter	CLEAR	SMILE	*P* value^a^	*P* value^b^	*P* value^c^
Preoperative					
Total area (µm^2^)	223.78 ± 135.29	202.46 ± 115.24	0.525	–	–
Perimeter (µm)	22.20 ± 7.02	21.44 ± 5.97	0.859	–	–
Postoperative month 1					
Total area (µm^2^)	290.73 ± 163.79	337.50 ± 237.15	0.315	**0.035**	**0.011**
Perimeter (µm)	30.54 ± 6.28	28.22 ± 8.64	0.695	**0.048**	**0.018**
Postoperative month 3					
Total area (µm^2^)	256.97 ± 133.82	239. 00 ± 170.59	0.248	0.584	0.537
Perimeter (µm)	26.34 ± 7.54	25.36 ± 6.36	0.569	0.323	0.052
Postoperative month 6					
Total area (µm^2^)	246.27 ± 142.03	221.84 ± 110.21	0.891	0.436	0.318
Perimeter (µm)	26.13 ± 7.32	25.27 ± 7.21	0.384	0.370	0.252
Postoperative month 12					
Total area (µm^2^)	246.70 ± 186.32	222.79 ± 153.66	0.708	0.965	0.190
Perimeter (µm)	25.63 ± 7.33	25.03 ± 5.77	0.333	0.895	0.361

*P* values in bold indicate statistical significance

*CLEAR* = corneal lenticule extraction for advanced refractive correction; *SMILE* = small incision lenticule extraction

^a^CLEAR vs. SMILE eyes

^b^Postoperative vs. preoperative for CLEAR eyes

^c^Postoperative vs. preoperative for SMILE eyes

### Corneal epithelial cell parameters

There were no significant differences in any of the corneal epithelial cell parameters between the CLEAR and SMILE groups at any time point. Compared to preoperative measurements, there were also no significant changes in all the epithelial parameters in all the eyes throughout the study period (Supplementary Table 2).

### Tear neuromediator changes following CLEAR and SMILE

**Fig. 5:**
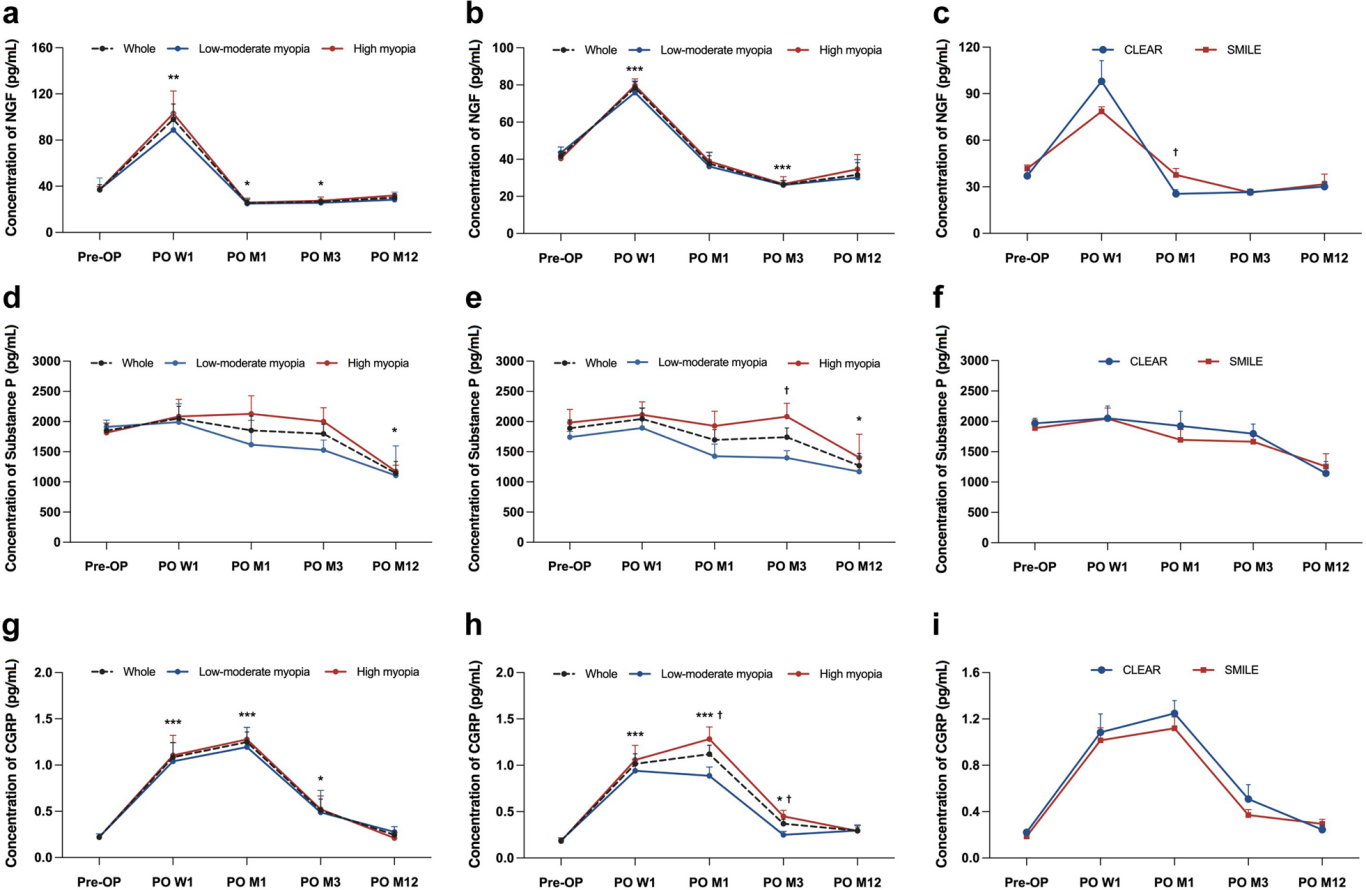
Changes of tear neuromediator concentrations at different time points. Tear nerve growth factor (NGF) concentrations in CLEAR eyes (**a**), SMILE eyes (**b**), and the comparison between CLEAR and SMILE (**c**); tear substance P concentrations in CLEAR eyes (**d**), SMILE eyes (**e**), and the comparison between CLEAR and SMILE (**f**); and tear calcitonin gene-related peptide (CGRP) concentrations in CLEAR eyes (**g**), SMILE eyes (**h**), and the comparison between CLEAR and SMILE (**i**). * indicates the comparison with preoperative levels and ^**†**^ indicates the comparison between two groups. *^,†^*P* < 0.05, **P < 0.01, ***P < 0.001. CLEAR, corneal lenticule extraction for advanced refractive correction; SMILE, small incision lenticule extraction; Pre-OP, preoperative; PO, postoperative; W, week; M, month

After CLEAR surgery, the tear NGF concentration peaked at 1 week (97.98 ± 49.76 pg/mL vs. preoperative 37.06 ± 17.60 pg/mL, *P* = 0.001), and then decreased to a significantly lower concentration at 1 month (24.35 ± 1.15 pg/mL, *P* = 0.024) and 3 months (26.58 ± 7.75 pg/mL, *P* = 0.024; Fig. 5a). Post-SMILE eyes showed a similar trend, peaking at 1 week (78.57 ± 10.77 pg/mL, *P* < 0.001) and significantly decreasing at 3 months (26.31 ± 7.32 pg/mL, *P* < 0.001; Fig. 4b). We further categorized the eyes into low-moderate treatment (corrected SE less than − 6.00 D; n = 8 eyes) and high myopia treatment (corrected SE greater than − 6.00 D; n = 11 eyes) groups. For both procedures, the high myopia group showed a slightly higher but non-significant NGF concentration than the low-moderate myopia group at all time points (Fig. 5a, b). At 1 month, the NGF concentrations of SMILE eyes were significantly higher than those with CLEAR eyes (37.66 ± 16.69 pg/mL vs. 24.35 ± 11.15 pg/mL, *P* = 0.013; Fig. 5c).

The concentrations of substance P and CGRP exhibited no significant differences between both procedures at all timepoints. Tear substance P concentration remained stable from 1 week to 3 months and significantly decreased at 12 months in both groups, with the concentration at 1144.41 ± 513.98 pg/mL in the CLEAR eyes (*P* = 0.041), and 1257.04 ± 740.00 pg/mL (*P* = 0.045) in the SMILE eyes (Fig. 5d, e). In the SMILE eyes, the concentration of substance P was significantly higher in the high myopia group compared to the low-moderate myopia group at 3 months (1977.20 ± 569.07 pg/mL vs. 1398.97 ± 315.79 pg/mL, *P* = 0.041) and no such significant difference was observed in the CLEAR eyes (Fig. 5d, e). After CLEAR, CGRP significantly increased at 1 week (1.09 ± 0.57 ng/mL, *P* < 0.001), 1 month (1.25 ± 0.45 ng/mL, *P* < 0.001) and 3 months (1.25 ± 0.45 ng/mL, *P* = 0.042) than preoperative levels (0.22 ± 0.08 ng/mL; Fig. 5g) without significant difference between the high and the low-moderate myopia group at all time points. Similarly, the CGRP concentration in post-SMILE eyes significantly increased at 1 week (1.02 ± 0.40 ng/mL, *P* < 0.001), 1 month (1.12 ± 0.40 ng/mL, *P* < 0.001), and 3 months (0.37 ± 0.18 ng/mL, *P* = 0.031), compared to that preoperatively (0.19 ± 0.08 ng/mL; Fig. 5h). The tear CGRP concentration in the high myopia group was higher than those in the low-moderate myopia group at all time points, and the differences were statistically significant at 1 month (*P* = 0.039) and 3 months (*P* = 0.034; Fig. 5h).

## Discussion

In this RCT, we investigated corneal denervation and subsequent regeneration, tear neuro-mediator profiles, and ocular surface changes following CLEAR and SMILE over a course of 1 year. The RCT and paired-eye design eliminates inter-individual variation and selection bias, allowing for more accurate comparisons. We demonstrated that CLEAR and SMILE led to comparable reduction of corneal nerve metrics and the resultant postoperative dry eye. In both procedures, the extent of nerve reduction was associated with the corrected SE and did not return to preoperative levels at 1 year. Tear NGF concentration was significantly higher in SMILE than CLEAR at the postoperative 1 month timepoint.

In KLEx surgery, several factors are related to the extent of corneal nerve damage: (1) Lenticule profile, as the lenticule extraction removes a portion of corneal nerve fibers. CLEAR and SMILE differ in lenticule geometry. The Femto LDV Z8 uses a flat contact glass in the lenticule cutting under applanation, creating a planoconvex lenticule that assumes a convex-concave shape with a tapered edge when the cornea is relaxed. The Visumax system employs a curved cone, creating a convex-concave lenticule with a 10–30 µm side cut based on the correction power [1]; (2) Cap thickness and optical zone. A larger optical zone implies a shorter length of peripheral nerve fibers being preserved, while a greater cap thickness preserves more superficial nerves [29]. The cap thickness in CLEAR and SMILE had a slight discrepancy in one case in our study due to the consideration of multiple factors, such as the patient's total corneal thickness, the refractive error to be corrected, corneal biomechanics, optical zone, and residual stromal bed thickness. However, previous studies have demonstrated comparable corneal nerve metrics after SMILE across different cap thicknesses, including 100 μm vs. 120 μm [30], 110 μm, 120 μm, and 130 μm [29], as well as 110 μm vs. 150 μm [31]. Therefore, the potential impact of cap thickness variation on corneal nerve change is expected to be minimal; (3) Incision size and number, as the subbasal nerve fibers are directly truncated. In the present study, CLEAR and SMILE employed a single incision of 2.1 mm, identical optical zone diameter and comparable cap thickness for the same patient. Additionally, the total suction time was comparable for both procedures at approximately 30 s. This consistent control of surgical parameters eliminates potential confounders; (4) Programmed SE. A comparable programmed SE is expected to cause similar stromal tissue removal and nerve interference according to Munnerlyn’s formula [32]. Taking into account the above, these collectively explain our study findings that there was no significant difference in postoperative denervation between CLEAR and SMILE. Our findings suggest that during the decision making between these two procedures, the impact on corneal denervation will not be the first consideration as they are comparable. Furthermore, it is important to note that the results of our study are based on a single-incision CLEAR approach, which is commonly preferred by experienced surgeons [9, 33]. Creating two incisions during the CLEAR procedure may lead to increased corneal nerve damage, and future studies may include comparisons of corneal denervation between one and two incisions.

At 1 year postoperatively, corneal nerve metrics in both the CLEAR and SMILE eyes had not recovered to the preoperative levels. The CNFD, CNBD, and CNFL, levels at 1 year were at 62.8%, 50.6%, and 77.1% of preoperative levels in the CLEAR eyes, and were at 58.7%, 43.3%, and 65.2% of preoperative levels in the SMILE eyes, respectively. These findings are in line with our previous study, showing that even 5 years after SMILE, CNFD and CNBD still remained at 62.8% and 78.0% of normal levels, respectively [4].

In both CLEAR and SMILE, the reduction in corneal nerve parameters was significantly and negatively correlated with the corrected SE. This finding aligns with our previous study, showing a significant and negative correlation between the corrected refractive power and the reduction in CNFD, CNBD, CNFL, CNFA, and CFracDim after SMILE [24]. Similarly, it has been reported that patients who underwent high myopic SMILE had a significantly higher reduction in CNFD than those who underwent low-moderate myopic SMILE [16].

At the site of nerve axonal injury, the Schwann cell tube directs the sprouting of axons from the proximal segment [34]. This leads to the regeneration of the injured axons and the formation of microneuromas, which are recognized as dynamic markers of corneal neuropathological recovery [35]. The morphological changes in microneuromas following CLEAR and SMILE show similar trends, with a significant increase in the total area and perimeter of microneuromas at one month, indicating high repair and nerve regeneration activities. Corneal microneuromas can still be observed 5 years after SMILE, suggesting that postoperative corneal nerve regeneration is a long drawn out process [4].

Dry eye symptoms following refractive surgery are primarily caused by corneal nerve damage and inflammation [36]. The flapless nature and small incision in CLEAR and SMILE preserve a greater proportion of corneal nerves, which in turn better preserves cornea-blink reflex, tear production loop, and tear film stability [37]. The disruption to corneal epithelial cells was also minimized in both procedures, as demonstrated by our corneal epithelial cell analysis. The changes in DCs demonstrated a comparable corneal inflammatory response following both procedures. Additionally, the suction system can affect the ocular surface outcome. Unlike the VisuMax, which employs a curved cone and limbal suction, the Femto LDV uses a flat interface cone and more posterior limbal/bulbar conjunctival suction. Although the latter may cause more pressure on the ocular surface, the ocular surface changes showed no significant difference. Here, all the ocular surface assessments presented were comparable between CLEAR and SMILE eyes at all time points as the extent of corneal denervation was similar. TBUT significantly decreased at 1 week and 1 month in both procedures, with a significant correlation with CNFL. This can be attributed to postoperative ocular surface inflammation [38], corneal denervation that decreases tear secretion and disrupts the function of goblet cells [39], and the ocular surface disruptions affecting mucin absorption and distribution [40]. The Schirmer's test values showed a progressive decrease at 6 and 12 months postoperatively compared to preoperative levels in both procedures. However, these differences were not significant, which was in agreement with previous studies [41, 42]. We also found that Schirmer’s values significantly correlated with CNFD, CNFL, CTBD, CNFA, and CFracDim, suggesting that tear production following CLEAR and SMILE are affected by alterations in corneal nerves.

Surgical incisions and laser exposure activate stromal keratocytes and trigger neuroinflammation [43]. The laser energy profile and the shape of the lenticule are associated with neuroinflammation. In both CLEAR and SMILE, the corneal epithelium, Bowman’s layer, and anterior stroma are injured due to the creation of the lenticule and incision [44]. Moreover, corneal nerves at the incision site and within the refractive lenticule are disrupted, whereas nerve bundles outside the cap or lenticule area remain untouched. Furthermore, previous studies have emphasized the relationship between postoperative inflammatory responses and the energy level of the laser used, with lower-energy femtosecond lasers significantly having less inflammatory reactions and minimizing cell death in tissues adjacent to the laser-treated area [45]. NGF is a well-characterized neurotrophin essential for maintaining corneal nerve density and sensation [46]. Our results indicated that tear NGF concentrations following both procedures peaked at 1 week and returned to near or below preoperative concentrations by 1 month, aligning with previous findings that tear NGF concentrations increased 1 week after SMILE and gradually decreased to significantly below preoperative levels at 6 and 12 months [16]. These findings can be explained by the substantial release of NGF due to active corneal neuroinflammation in the early postoperative period, which gradually depletes and declines as neuroregeneration progresses. Furthermore, SMILE eyes had significantly higher tear NGF concentration than CLEAR eyes at 1 month, suggesting greater neuroinflammation, but warrants further investigation.

CGRP is a multifunctional neuropeptide constitutively expressed in tears [47]. Its secretion increases during corneal epithelial injury to accelerate re-epithelialization [48], with elevated concentrations correlating with more severe dry eye symptoms [49]. We found that tear CGRP concentrations significantly increased in both CLEAR and SMILE eyes within 1 month and then returned to preoperative levels, while increased tear CGRP concentrations was noted to last for 1 year after LASIK [43], demonstrating the less invasiveness and neuroinflammatory reaction of CLEAR and SMILE compared to LASIK [50]. Substance P is another highly abundant neuropeptide that primary triggers neurogenic inflammation [51]. In our study, tear substance P concentrations remained unchanged during the first 3 months after CLEAR and SMILE but decreased at 12 months, consistent with the study by Chin et al. [16]. The minimal disruptions of the ocular surface in CLEAR and SMILE may allow substance P concentrations to remain stable during the initial postoperative phase, with subsequent decreases indicating depletion of this neuropeptide as corneal healing progresses.

Additionally, high myopic correction led to significantly higher tear CGRP and substance P concentrations than low-moderate myopic correction at 1 and 3 months in SMILE, but not in CLEAR. Riau et al. previously demonstrated that the low-energy, high-frequency LDV system led to fewer corneal apoptotic cells and lesser wound healing compared to the VisuMax system [1], which employs higher laser pulse energy and a higher repetition rate, supporting and explaining our findings [52].

The study has a relatively small sample size but was sufficiently powered to detect the difference in tear neuromediator profiles between both procedures. As both CLEAR and SMILE are lenticule-based, one would not expect to see a significant difference in the postoperative corneal nerve parameters. Furthermore, given the minimal difference in corneal nerve metrics between the procedures, the required sample size will be 4552 participants to observe the difference in the corneal denervation. While statistically valid, this would be clinically irrelevant. Our study involved slight differences in the cap thickness in one case and incision positioning between the two procedures. While such variations are unlikely to lead to a significant impact on corneal nerve changes and related ocular surface outcomes, future studies with identical incision positioning between both procedures will better eliminate all the potential bias. Future studies with larger cohorts are warranted for subgroup analyses, such as the comparisons among different cap thicknesses. Additionally, this study did not include subjective symptom evaluation using questionnaires such as the ocular surface disease index since these assess symptoms at the individual level rather than at the eye level, making it difficult to determine whether postoperative ocular discomfort was attributable to SMILE or CLEAR.

## Conclusions

In conclusion, to our knowledge, this is the first study to assess CLEAR from the aspects of corneal denervation and biological responses. Our RCT data indicates that CLEAR and SMILE procedures do not differ in the impact on corneal denervation and subsequent regeneration, as well as clinical dry eye outcomes. Corneal nerve metrics did not restore even at 1 year for both procedures. The postoperative neuroinflammation following CLEAR and SMILE were generally comparable, although tear NGF concentration was higher in SMILE at the 1 month timepoint. These findings offer valuable insights into the corneal nerve and neuroinflammation features after CLEAR and SMILE, enhancing the understanding of postoperative effects on the ocular surface and the underlying pathophysiological changes from both morphological and molecular perspectives.

## Supplementary Information

Supplementary Material 1.

## Data Availability

The original contributions presented in this study are included in this article/supplementary material, further inquiries can be directed to the corresponding authors.

## Abbreviations

KLEx: Keratorefractive lenticule extraction
SMILE: Small incision lenticule extraction
LASIK: Laser-assisted in situ keratomileusis
CLEAR: Corneal lenticule extraction for advanced refractive correction
UCVA: Uncorrected distance visual acuity
CGRP: Calcitonin gene-related peptide
NGF: Nerve growth factor
RCT: Randomized controlled trial
MRSE: Manifest refractive spherical equivalent
NEI: National Eye Institute
TBUT: Tear break-up time
IVCM: In vivo confocal microscopy
CNFD: Corneal nerve fiber density
CNFL: Corneal nerve fiber length
CNBD: Corneal nerve branch density
CTBD: Corneal nerve total branch density
CNFA: Corneal nerve fiber area
CNFW: Corneal nerve fiber width
CFracDim: Corneal nerve fiber fractal dimension
DCs: Dendritic cells
BCVA: Best-correct visual acuity
