# Supplementary material for: Randomized controlled trial on corneal denervation, neuroinflammation and ocular surface in corneal lenticule extraction for advanced refractive correction (CLEAR) and small incision lenticule extraction (SMILE)
Source: Eye Vis (Lond). 2025 Apr 1;12:12. doi: 10.1186/s40662-025-00429-1 (PMC11959778; doi:10.1186/s40662-025-00429-1)
Supplement: Supplementary file 1 — Supplementary Material 1. [file 40662_2025_429_MOESM1_ESM.docx]

| **Inclusion criteria** |
| --- |
| 1. Cycloplegic spherical equivalent of greater than −1.00 D, refractive cylinder −2.00 D or less, and anisometropia less than 1.00 D. 2. Best spectacle corrected visual acuity (BSCVA) of 6/12 or better in both eyes. 3. Contact lens removed at least 2 weeks before the baseline measurement. 4. No evidence of irregular astigmatism on corneal topography. |
| **Exclusion criteria** |
| 1. Progressive or unstable myopia and/or astigmatism. 2. Clinical or corneal topographic evidence of keratoconus. 3. Residual, recurrent, or active ocular disease such as uveitis. 4. Severe dry eyes, severe allergic eye disease, glaucoma, visually significant cataract, and retinal disease. 5. Previous corneal surgery or trauma within the corneal treatment zone. 6. Corneal vascularization within 1mm of the corneal treatment zone. 7. Taking systemic medications that may affect tear neuromediator analysis or wound healing, such as corticosteroids and antimetabolites. 8. Systemically immunocompromised. 9. Systemic disease likely to affect wound healing, such as diabetes, connective tissue disease and severe atopy. |

**Supplementary Table 1.** Inclusion and exclusion criteria for the study population.

**Supplementary Table 2.** Comparison of corneal epithelial cell parameters in CLEAR vs. SMILE.

| **Parameter** | **CLEAR** | **SMILE** | ***P* value^a^** | ***P* value^b^** | ***P* value^c^** |
| --- | --- | --- | --- | --- | --- |
| Preoperative |  |  |  |  |  |
| Circularity | 0.73 ± 0.01 | 0.74 ± 0.01 | 0.599 | - | - |
| Cell density (/um^2^) | 0.0076 ± 0.0001 | 0.0073 ± 0.0005 | 0.514 | - | - |
| Average size (um) | 132.81 ± 1.12 | 138.09 ± 9.24 | 0.500 | - | - |
| Postoperative month 1 |  |  |  |  |  |
| Circularity | 0.73 ± 0.00 | 0.73 ± 0.013 | 0.385 | 0.686 | 0.144 |
| Cell density (/um^2^) | 0.0074 ± 0.0013 | 0.0076 ± 0.0005 | 0.812 | 0.139 | 0.951 |
| Average size (um) | 138.44 ± 22.51 | 132.33 ± 8.99 | 0.685 | 0.146 | 0.937 |
| Postoperative month 3 |  |  |  |  |  |
| Circularity | 0.72 ± 0.00 | 0.71 ± 0.01 | 0.098 | 0.417 | 0.098 |
| Cell density (/um^2^) | 0.0086 ± 0.0012 | 0.0081 ± 0.0015 | 0.662 | 0.070 | 0.816 |
| Average size (um) | 119.04 ± 14.79 | 131.39 ± 15.18 | 0.260 | 0.080 | 0.787 |
| Postoperative month 6 |  |  |  |  |  |
| Circularity | 0.71 ± 0.02 | 0.72 ± 0.01 | 0.590 | 0.287 | 0.077 |
| Cell density (/um^2^) | 0.0090 ± 0.0012 | 0.0089 ± 0.0013 | 0.912 | 0.189 | 0.115 |
| Average Size (um) | 121.82 ± 13.32 | 121.19 ± 11.26 | 0.945 | 0.339 | 0.089 |
| Postoperative month 12 |  |  |  |  |  |
| Circularity | 0.72 ± 0.01 | 0.70 ± 0.02 | 0.313 | 0.272 | 0.072 |
| Cell density (/um^2^) | 0.0082 ± 0.0010 | 0.0081 ± 0.0006 | 0.828 | 0.387 | 0.114 |
| Average size (um) | 123.06 ± 15.82 | 123.92 ± 9.47 | 0.927 | 0.448 | 0.105 |

*CLEAR* = corneal lenticule extraction for advanced refractive correction; *SMILE* = small incision lenticule extraction

^a^ CLEAR vs. SMILE eyes.

^b^ Postoperative vs. preoperative for CLEAR eyes.

^c^ Postoperative vs. preoperative for SMILE eyes.
